# Trends in incidence and case fatality of acute myocardial infarction, angina and coronary revascularisation in people with and without type 2 diabetes in Scotland between 2006 and 2015

**DOI:** 10.1007/s00125-018-4796-7

**Published:** 2019-01-17

**Authors:** Stephanie H. Read, Colin M. Fischbacher, Helen M. Colhoun, Danijela Gasevic, Joannes J. Kerssens, David A. McAllister, Naveed Sattar, Sarah H. Wild, on behalf of the Scottish Diabetes Research Network Epidemiology Group

**Affiliations:** 1 Usher Institute of Population Health Sciences and Informatics, University of Edinburgh, Teviot Place, Edinburgh, EH8 9AG UK; 2 Women’s College Research Institute, Women’s College Hospital, Toronto, ON Canada; 3 Information Services Division, NHS National Services Scotland, Edinburgh, UK; 4 Institute of Genetics and Molecular Medicine, University of Edinburgh, Western General Hospital, Edinburgh, UK; 5 School of Public Health and Preventive Medicine, Monash University, Melbourne, VIC Australia; 6 Institute of Health and Wellbeing, University of Glasgow, Glasgow, UK; 7 Institute of Cardiovascular and Medical Sciences, University of Glasgow, Glasgow, UK

**Keywords:** Angina, Coronary heart disease, Myocardial infarction, Revascularisation, Type 2 diabetes

## Abstract

**Aims/hypothesis:**

The aim of the study was to examine trends in the incidence and case fatality of acute myocardial infarction (AMI) and in hospital admissions for angina and coronary revascularisation procedures in people with type 2 diabetes and in people without diabetes in Scotland between 2006 and 2015.

**Methods:**

In this retrospective cohort study, AMI, angina and revascularisation event data were obtained for adults from hospital admissions and death records linked to a population-based diabetes register. Incidence by diabetes status was estimated using negative binomial models with adjustment or stratification by age, sex, deprivation and calendar year. Logistic regression was used to estimate AMI case fatality by diabetes status.

**Results:**

There were 129,926 incident AMI events, 41,263 angina admissions and 69,875 coronary revascularisation procedures carried out during 34.9 million person-years of follow-up. The adjusted incidence of AMI, angina and revascularisation procedures declined by 2.0% (95% CI 1.73%, 2.26%), 9.62% (95% CI 9.22%, 10.01%) and 0.35% (95% CI −0.09%, 0.79%) per year, respectively. The rate of decline did not differ materially by diabetes status. RRs of AMI for type 2 diabetes were 1.86 (95% CI 1.74, 1.98) for men and 2.32 (95% CI 2.15, 2.51) for women. Of the 77,211 people admitted to hospital with a first AMI, 7842 (10.2%) died within 30 days of admission. Case fatality was higher in people with type 2 diabetes than in people without diabetes and declined in both groups by 7.93% (95% CI 7.03%, 8.82%) per year.

**Conclusions/interpretation:**

The incidence of AMI, angina, revascularisation and AMI case fatality has declined over time, but the increased risk associated with type 2 diabetes has remained approximately constant.

**Electronic supplementary material:**

The online version of this article (10.1007/s00125-018-4796-7) contains peer-reviewed but unedited supplementary material, which is available to authorised users.

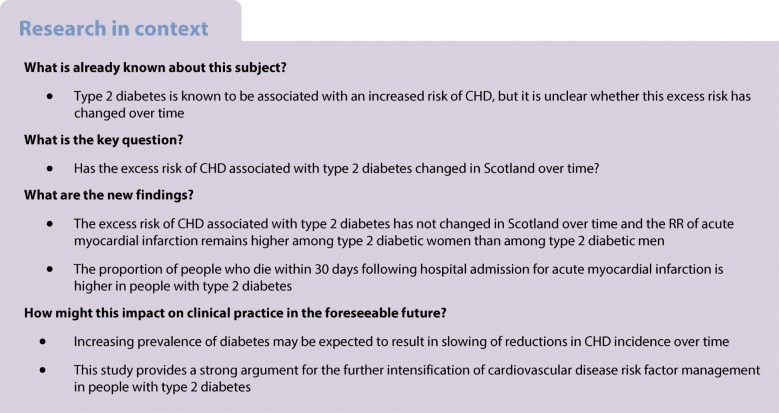



## Introduction

Type 2 diabetes is known to be associated with an elevated risk of cardiovascular disease (CVD) morbidity and mortality. In particular, people with type 2 diabetes have previously been shown to have up to a fivefold higher incidence of acute myocardial infarction (AMI) compared with people without diabetes [1–4].

Downward trends in the incidence of AMI have occurred in recent decades among people without diabetes in high income countries, partly due to improvements in the prevention and treatment of CVD [5]. Changes in the investigation of AMI, in the form of more sensitive tests and lower diagnostic thresholds, and increasing use of revascularisation procedures, might also have had different effects on patterns of hospital admission for CVD including AMI, angina and revascularisation in people with and without diabetes. Moreover, there have been considerable efforts to reduce the risk of CVD specifically in people with type 2 diabetes, through more intensive risk factor control and newer diabetes treatments purported to confer specific cardiovascular benefits [6, 7]. It is not known whether these approaches have had an effect on the higher risk of CHD conferred by type 2 diabetes. Consequently, there is a need for information on trends in the incidence of AMI, angina and procedures for CHD in people with diabetes. Scotland’s large representative whole population-linked datasets provide an opportunity to address this question. We examined trends in the incidence and case fatality of AMI and in hospital admissions for angina and revascularisation procedures in the Scottish adult population with type 2 diabetes compared with the non-diabetic population over the period 2006–2015.

## Methods

### Data sources

Data for the analyses were obtained from three sources. Mid-year population estimates by age, sex, calendar year and deciles of the 2012 Scottish Index of Multiple Deprivation (SIMD) were obtained from National Records of Scotland.

Data on type 2 diabetes diagnoses were obtained from the Scottish Care Information—Diabetes (SCI-Diabetes) dataset [8]. This dynamic clinical register was established in 2000; from 2006 onwards, coverage of the register was estimated to exceed 95% and is now above 99.5%. Diabetes type was defined using an algorithm which utilises age at diagnosis, prescriptions data and clinically assigned type of diabetes [9]. Deprivation was measured using the SIMD, an area-based measure of deprivation which uses information from seven domains including unemployment rates, crime and income to assign a deprivation score to 6976 small data zones in Scotland [10]. The data zones are ranked and grouped into deciles.

Event data were obtained from the Scottish Morbidity Records (SMR01) database, which captures all hospital admissions in Scotland, and from national death records. The primary endpoint was AMI, defined as a first hospital admission with a stated clinical diagnosis of AMI or CHD death, identified using ICD-10 codes I21-22 or I20-I25, respectively (http://apps.who.int/classifications/icd10/browse/2016/en). Secondary endpoints included incidence of hospital admissions or death due to angina (I20) and incidence of revascularisation procedures, identified using the fourth revision of the Office of Population Censuses and Surveys Classification of Interventions and Procedures: K40-K50, K75. Where there were multiple hospital episodes for a single incident event, the chronologically earliest episode was used. People with a history of the endpoints prior to entry to the cohort (based on date of diagnosis of diabetes and age and calendar time points, as appropriate) or who had incomplete deprivation data were excluded from the analyses. Case fatality was defined as death within 30 days following hospital admission with AMI. SCI-Diabetes, the SMR01 and death registrations were linked using the Community Health Index, a unique patient identifier, and probabilistic linkage methods [11]. Data linkage is well established in Scotland and, thanks to the widespread use of the Community Health Index in medical records, over 98% of records are able to be linked [12]. Generation of the anonymised linked dataset was approved by the Scotland Multi-Centre Research Ethics Committee (reference 11-AL-0225), Caldicott Guardians and the National Health Service (NHS) National Services Scotland Privacy Application Committee (reference 33/11); individual informed consent is not required for the linkage.

### Statistical analyses

Analyses were restricted to people aged between 30 and 89 years at any time during the study period, because of the small numbers of events outside this age range. The study period was between 1 January 2006 and 31 December 2015. Follow-up was from study start date for people with prevalent diabetes at this point or from date of diagnosis of diabetes, if between 2006 and 2015, until date of death, date of incident event or study end date, whichever came first. To calculate incident events and person-years in the non-diabetic comparison group, the number of incident events and person-years at risk for the population of people with any type of diabetes was subtracted from the number of events and the mid-year population estimates for the whole population, respectively. All analyses were conducted separately for men and women.

The AER package in R (R Foundation for Statistical Computing, Vienna, Austria) was used to apply Cameron and Trivedi’s test for over-dispersion [13, 14]. As there was some evidence of over-dispersion in the datasets (electronic supplementary material [ESM] Table 1), negative binomial regression models, stratified by sex and diabetes status, adjusted for age, calendar year and deciles of SIMD, were used to calculate standardised incidence rates and RRs for AMI, angina and revascularisation. An interaction term between diabetes status and deprivation was included in the models, since deprivation has previously been shown to modify the relationship between type 2 diabetes and CVD [15]. An interaction term between diabetes status and calendar year was also included to examine whether the association between type 2 diabetes and risk of AMI had changed over time. Other interaction terms between year, diabetes type, sex and deprivation were included where RRs associated with the interaction were <0.95 or >1.05 (ESM Table 2). Logistic regression models adjusted for age, sex, calendar year and deprivation were used to estimate the odds of case fatality following hospital admission with AMI associated with type 2 diabetes. All analyses were conducted in R, version 3.2.2.

## Results

Overall, 129,926 AMI events, 41,263 angina admissions and 69,875 coronary revascularisations were recorded during 34.9 million person-years of follow-up between 2006 and 2015 in Scotland. Of the AMI events, 24,390 (18.8%) occurred among people with type 2 diabetes. The crude incidence of AMI was 3.21 and 11.75 per 1000 person-years among people without diabetes and with type 2 diabetes, respectively. The proportion of AMI events that were fatal prior to hospital admission was higher in men with type 2 diabetes than in men without diabetes, but proportions were similar by diabetes status in women (Table 1).

**Table 1:** Number of events by type of CHD, timing relative to admission for AMI, diabetes status and sex

CHD type	Deaths before hospital admission (% of AMI events)	Total hospital admissions (% of AMI events)	Case fatality (% of AMI admissions)
AMI event (AMI or CHD death)			
Men			
No diabetes	25,134 (38.3)	40,477 (61.7)	3171 (7.8)
Type 2 diabetes	6645 (44.0)	8448 (56.0)	987 (11.7)
Women			
No diabetes	16,853 (42.2)	23,072 (57.8)	2897 (12.6)
Type 2 diabetes	4083 (43.9)	5214 (56.1)	787 (15.1)
Angina			
Men			
No diabetes		18,584	–
Type 2 diabetes		4725	–
Women			
No diabetes		14,809	–
Type 2 diabetes		3145	–
Coronary revascularisation			
Men			
No diabetes	–	42,497	–
Type 2 diabetes	–	8382	–
Women			
No diabetes	–	15,770	–
Type 2 diabetes	–	3226	–

### Incidence

**Fig. 1:**
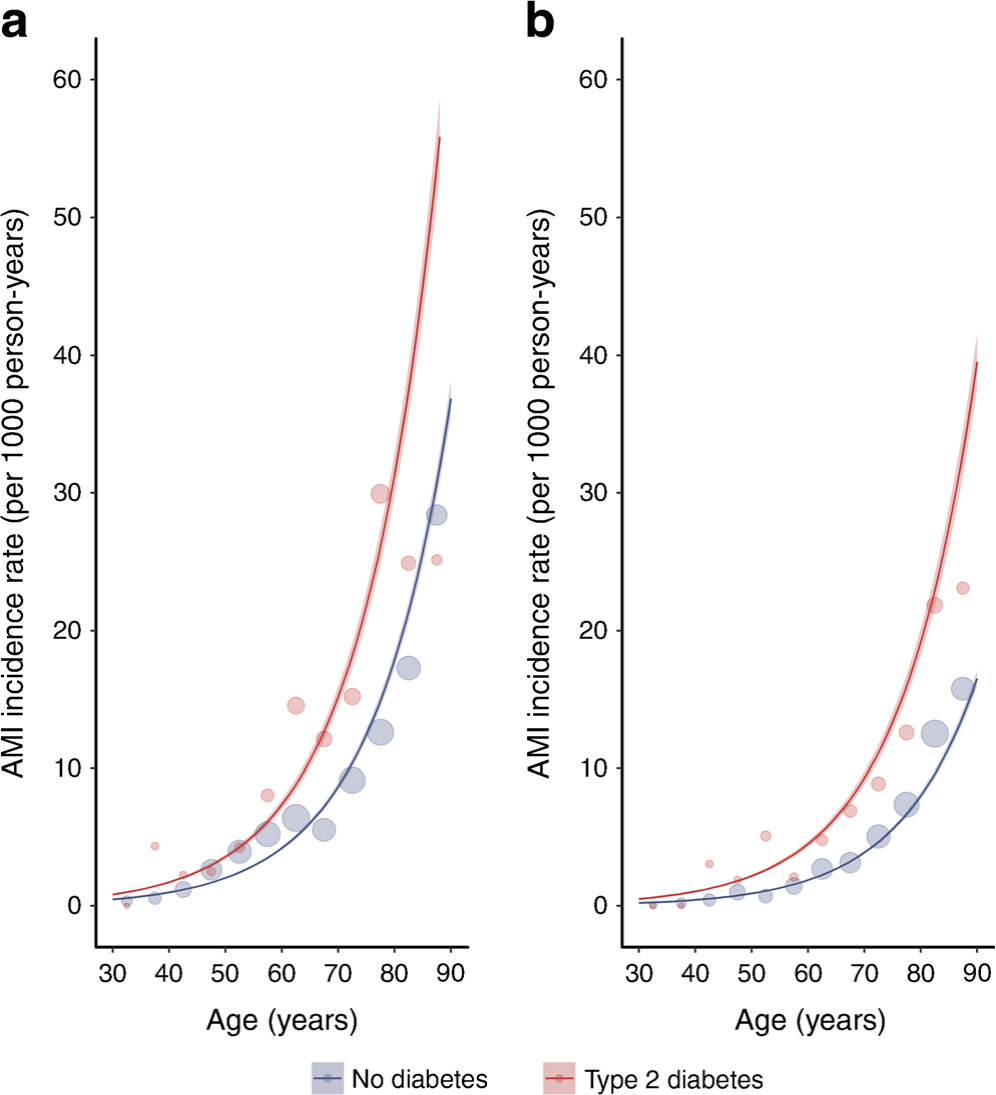
Incidence of AMI by diabetes status, sex and age in years. The graphs illustrate modelled rates for (**a**) men and (**b**) women in deprivation decile 5 in the calendar year 2010 using negative binomial models. The shading shows 95% CIs. Points represent event rates stratified by age in 5-year bands, sex and diabetes diagnosis; the size of the points reflects the size of the denominator

Figure 1 illustrates estimated AMI incidence by age and sex for people with type 2 diabetes and without diabetes from negative binomial models adjusted for calendar year and deprivation among people within deprivation quintile 5 in 2010. AMI incidence increased with age, was higher in men than in women, and was higher in people with type 2 diabetes than in people without diabetes. Overall, type 2 diabetes conferred a higher RR of AMI in women (2.32; 95% CI 2.15, 2.51) than in men (1.86; 95% CI 1.74, 1.98). Increasing deprivation was associated with AMI risk, which was 1.52 times (95% CI 1.50, 1.54) higher in the most deprived compared with the least deprived decile for men and 1.56 (95% CI 1.54, 1.58) times higher for women.

Similar patterns of higher incidence among people with type 2 diabetes compared with people without diabetes were observed for angina and coronary revascularisation (ESM Fig. 1, ESM Fig. 2).

### Time trends

The absolute numbers of events by calendar year, sex and diabetes status are presented in Table 2 and ESM Table 3. The proportion of people who had an incident AMI event and who had been diagnosed with type 2 diabetes increased from 19.2% in 2006 to 28.2% in 2015. The total number of AMI events increased between 2009 and 2010 alongside reductions in the number of angina admissions. The number of coronary revascularisations each year over the study period remained approximately constant in people without diabetes but increased in people with type 2 diabetes.

**Table 2:** Number of CHD events by calendar year, sex and diabetes status

CHD type	2006	2007	2008	2009	2010	2011	2012	2013	2014	2015	Overall
AMI or CHD death											
Men											
No diabetes	6487	6479	6532	6563	6936	6797	6787	6514	6290	6226	65,611
Type 2 diabetes	1218	1317	1343	1488	1597	1545	1676	1614	1568	1727	15,093
Events in men with type 2 diabetes, %	15.8	16.9	17.1	18.5	18.7	18.5	19.8	19.9	20.0	21.7	18.7
Women											
No diabetes	4398	4092	4126	4123	4184	4030	4101	3774	3616	3481	39,925
Type 2 diabetes	875	778	847	924	948	999	955	1026	934	1011	9297
Events in women with type 2 diabetes, %	16.6	16.0	17.0	18.3	18.5	19.9	18.9	21.4	20.5	22.5	18.9
Angina											
Men											
No diabetes	2440	2619	2534	2123	1854	1465	1435	1429	1378	1307	18,584
Type 2 diabetes	531	572	560	468	505	384	378	445	426	456	4725
Events in men with type 2 diabetes, %	17.9	17.9	18.1	18.1	21.4	20.8	20.8	23.7	23.6	25.9	20.3
Women											
No diabetes	2002	2064	2081	1664	1444	1153	1158	1157	1099	987	14,809
Type 2 diabetes	361	385	397	369	319	283	236	282	250	263	3145
Events in women with type 2 diabetes	15.3	15.7	16.0	18.2	18.1	19.7	16.9	19.6	18.5	21.0	17.5
Coronary revascularisation											
Men											
No diabetes	4065	4197	4007	4070	4255	4286	4395	4497	4424	4301	42,497
Type 2 diabetes	685	763	723	783	818	828	903	912	969	998	8382
Events in men with type 2 diabetes, %	14.4	15.4	15.3	16.1	16.1	16.2	17.0	16.9	18.0	18.8	16.5
Women											
No diabetes	1572	1635	1544	1479	1546	1574	1617	1628	1628	1547	15,770
Type 2 diabetes	295	268	258	337	316	338	287	361	376	390	3226
Events in women with type 2 diabetes, %	15.8	14.1	14.3	18.6	17.0	17.7	15.1	18.1	18.8	20.1	17.0

**Fig. 2:**
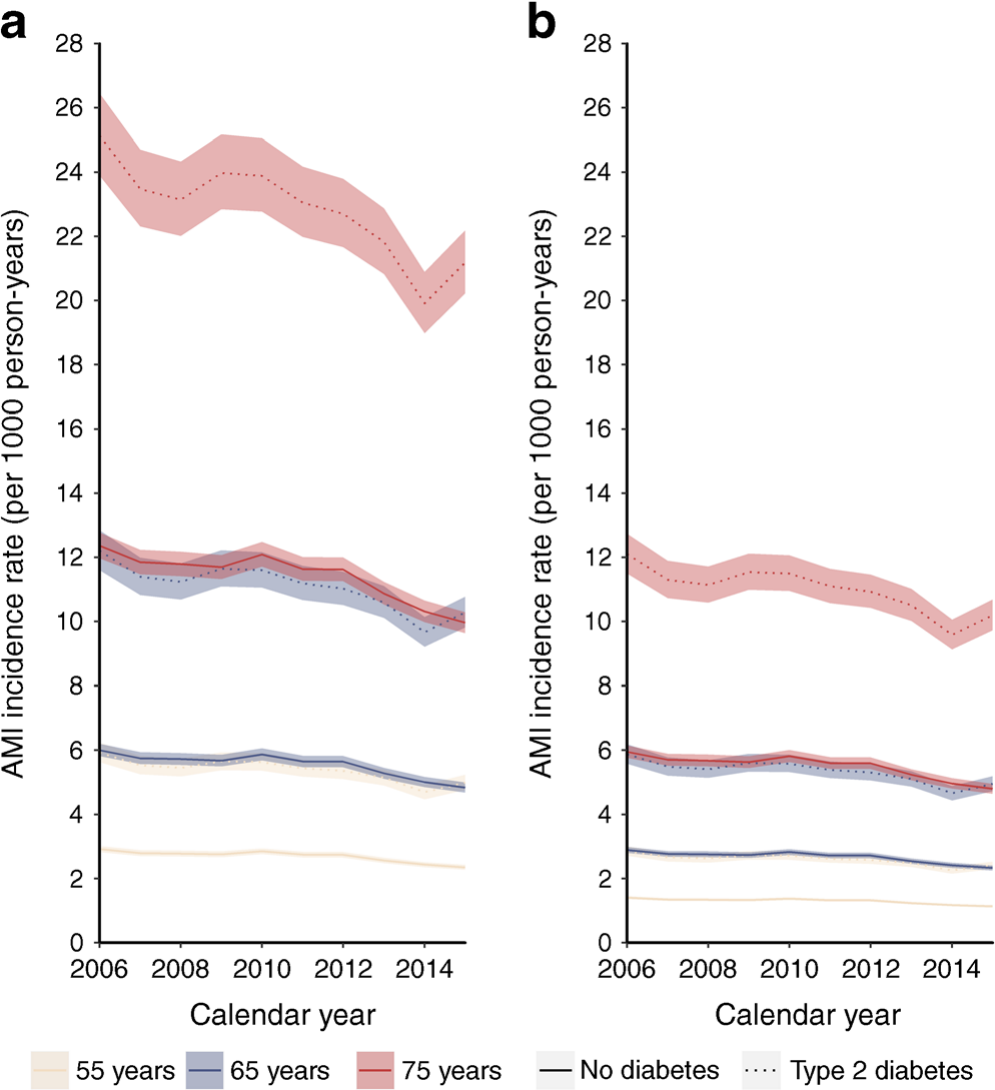
Trends in incidence of AMI by diabetes status and calendar year illustrated for (**a**) men and (**b**) women in deprivation decile 5 and aged 55, 65 and 75 years. Estimates derived from the model are adjusted for age, sex, deprivation and interaction terms between sex and year, sex and diabetes status, diabetes status and year. The line colour depicts the age and the line type depicts the diabetes status. The shading shows 95% CIs

Overall, after adjusting for age and deprivation, the incidence of AMI declined, on average, by 2.0% (95% CI 1.73%, 2.26%) per year (see example presented in Fig. 2) and there was no evidence that time trends differed appreciably by diabetes status (RR for year × diabetes interaction 1.01; 95% CI 1.00, 1.02). The incidence of angina and coronary revascularisation declined by 9.62% (95% CI 9.22%, 10.01%) and 0.35% (95% CI −0.09%, 0.79%), respectively (ESM Fig. 3, ESM Fig. 4). Declines in adjusted incidence of angina and revascularisation were also similar in people with type 2 diabetes and without diabetes (RRs for year × diabetes interactions: angina 1.01 [95% CI 1.00, 1.02]; revascularisation 1.00 [95% CI 0.99, 1.01]).

### Case fatality

**Fig. 3:**
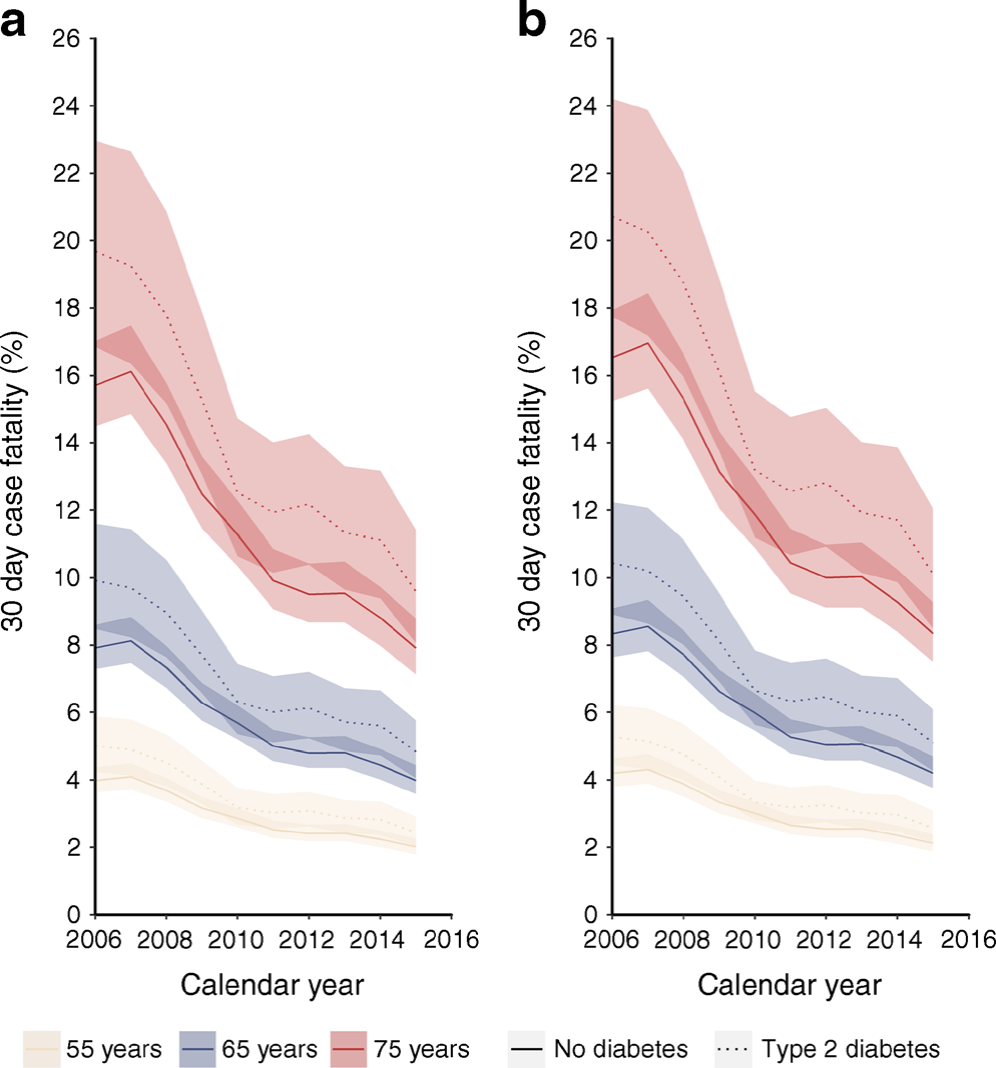
Case fatality within 30 days of hospital admission following AMI in (**a**) men and (**b**) women aged 55, 65 and 75 years, by calendar year and diabetes status. Estimates derived from model adjusted for age, sex, calendar year, deprivation status and interaction terms between sex and year, sex and diabetes status, deprivation and diabetes status, diabetes status and year. The line colour depicts the age and the line type depicts the diabetes status. The shading shows 95% CIs

Of the 77,211 people hospitalised due to AMI, 7842 (10.2%) died within 30 days of hospital admission. Case fatality was higher in people with type 2 diabetes than in people without diabetes (13% vs 9.5%) and higher in women compared with men (13% vs 8.5%), and it increased with increasing age (ESM Fig. 5). In adjusted logistic regression models, type 2 diabetes conferred a similar excess odds of case fatality in men (OR 1.22; 95% CI 1.13, 1.32) and women (OR 1.19; 95% CI 1.09, 1.30). Case fatality following AMI declined, on average, by 7.93% (95% CI 7.03%, 8.82%) per year during the study period, and the rate of decline was similar in people with type 2 diabetes and people without diabetes (RR for interaction 1.00; 95% CI 0.98, 1.02) (Fig. 3). The greatest declines in case fatality occurred in the earlier years of the study period.

## Discussion

In this population-based study, we report declining age- and deprivation-adjusted incidence of AMI, angina and coronary revascularisations in Scotland between 2006 and 2015, regardless of diabetes status. Despite improvements in absolute risk, the risk of AMI remained 1.9–2.3 times higher in people with type 2 diabetes compared with people without diabetes. This suggests that changes in diagnostic practice, measures to address cardiovascular risk factors and introduction of new treatments for diabetes in recent years have not had a discernible differential effect in people with type 2 diabetes compared with people without diabetes.

Similarly, 30 day case fatality following hospital admission with AMI improved over time both in people with type 2 diabetes and in people without diabetes but remained consistently higher in people with type 2 diabetes.

### Relation to other studies

Our findings of stable RRs of first AMI in both men and women with type 2 diabetes are in agreement with some [2, 16, 17] but not all [3, 18–20] earlier findings. In the UK, type 1 and type 2 diabetes conferred a four- to fivefold increased risk of AMI and this excess risk did not change significantly between 2004 and 2009; however, these estimates include recurrent events, so they are not directly comparable [2, 21]. By contrast, findings from the US National Health Interview Survey show that the RRs of AMI in people with diabetes compared with the non-diabetic population declined from 2.8 in 2000 to 1.8 in 2010 [3]. Similar reductions in incidence rate ratios for CHD in people with diabetes compared with the non-diabetic population were observed between 1998 and 2010 in Western Australia [19]. It is possible that these conflicting findings reflect differences in health services between populations.

Improvements in CHD incidence for people with and without diabetes in high income countries are likely to reflect population-wide reductions in smoking prevalence [22], reduced dietary salt intake [23, 24] and improved treatment of hypertension and dyslipidaemia. For example, smoking prevalence declined from 28% to 21% between 2003 and 2015 in the Scottish general population and from 21.4% to 18.1% between 2005 and 2015 in the diabetes population [25, 26]. The Quality and Outcomes Framework incentivised improved management of CVD risk in primary care among people with chronic diseases such as type 2 diabetes and was introduced across the UK in 2004. This might have been expected to result in more rapid declines in CVD risk in people with diabetes compared with the non-diabetic population, although better management of other risk factors such as hypertension was supported by the scheme regardless of diabetes status. Unfortunately, population-wide data for trends in AMI among people with type 2 diabetes prior to 2004 in Scotland were not available to enable us to describe earlier RR estimates. Nonetheless, our finding of unchanging RRs for CHD by diabetes status is perhaps surprising given the additional support for risk factor management and that the characteristics of people with newly diagnosed type 2 diabetes are likely to have changed in recent years [27]. Diagnosis of diabetes earlier in the disease might have been expected to result in faster declines in AMI incidence among people with type 2 diabetes than in people without diabetes [28]. However, we did not observe this pattern, suggesting that any such effects might have occurred prior to 2006, or that a longer period of follow-up is required before the effect of earlier diagnosis of diabetes can be observed at a population level. Furthermore, obesity prevalence increased by a greater extent in people with type 2 diabetes than in the general population in Scotland between 2005 and 2013, which, along with patterns of other risk factors such as physical inactivity that may have changed over the study period either differentially or non-differentially by diabetes status, might also have influenced our findings [25, 26].

While we report average annual declines of 2.0% in incidence of AMI, it is clear that AMI incidence increased slightly (and angina incidence fell) following the introduction of lowered troponin thresholds to diagnose AMI in 2008. The increase after 2008 was less marked in women, which may reflect the use of a single revised diagnostic threshold for men and women at this time. Subsequent validation of the newer high sensitivity troponin assay suggested that sex-specific thresholds were required to avoid underdiagnosis of AMI in women [29]. The introduction of more sensitive diagnostic tools for AMI is likely to have led to the diagnosis of less severe AMI, particularly among people who might have previously received a diagnosis of angina, and could partly account for the steep declines in 30 day case fatality following hospital admission with incident AMI and in the declines in angina incidence rates in more recent years. Unfortunately, we did not have access to data on the introduction of new assays and thresholds across Scotland.

Higher RRs of incident AMI for people with diabetes compared with people without diabetes and for women compared with men have been reported previously [18, 30]. In one meta-analysis, the RR of incident CHD associated with diabetes was 44% greater in women than in men [30]. Mechanisms for this sex disparity include differences in prevalence and management of cardiovascular risk factors by sex and diabetes status [31, 32]. However, in a recent meta-analysis including data for 980,793 people, the RRs of diabetes for vascular mortality remained much higher in women than in men even after adjustment for CVD risk factors [33].

### Strengths and weaknesses

Our estimates of trends in the incidence of first AMI, angina and coronary revascularisation are based on a complete national population and are therefore unlikely to be have been affected by selection bias. We were also able to identify people with a diagnosis of type 2 diabetes from the national diabetes register, rather than depending on hospital admission records in which diabetes is under-reported [33].

Unfortunately, the accuracy of recording of incident AMI events in routinely collected data may not be perfect. However, the data sources are audited annually and the recorded diagnosis was found to be correct for >95% of AMI events [34]; it seems unlikely that a discrepancy in accuracy of recording of AMI by diabetes status contributed to our findings. Similarly, we are not able to exclude the potential for misclassification of vital status if the proportions of people who die outside Scotland differ by diabetes status. We were unable to adjust estimates for important confounders including CVD risk factors, since we did not have access to these data for the general population. We were therefore unable to determine how risk factor patterns had changed over time or to what extent differences in risk factors contributed to the disparity in AMI incidence between people with type 2 diabetes and people without diabetes. Given that diabetes is associated with worse CVD risk factor profiles [35], it is fair to assume that the excess risk of CHD observed in this study was in part due to differences in the distribution of CVD risk factors between the groups. Our study was also limited by the inability to separate ST-elevation (STEMI) from non-ST-elevation (NSTEMI) AMI events.

## Conclusions

The incidence of AMI, angina and revascularisation procedures declined at similar rates in people with and without type 2 diabetes in Scotland between 2006 and 2015. Compared with people without diabetes, the risk of incident CHD remained considerably higher among people with type 2 diabetes, who also had higher 30 day case fatality following hospital admission with AMI. These findings have important implications for primary and secondary prevention of CHD in the future, given the growing prevalence of type 2 diabetes and an ageing population.

## Electronic supplementary material

ESM (PDF 741 kb).

## Data Availability

The data that support the findings of this study are available from the SCI-Diabetes database and the Electronic Data Research and Innovation Service of the Information Services Division of the NHS National Services Scotland, but restrictions apply to the availability of these data, which were used under licence for the current study and so are not publicly available. Data are, however, available with permission of the NHS Scotland Public Benefit and Privacy Panel for Health and Social Care. HMC reports grants, personal fees and non-financial support from AstraZeneca, Boehringer Ingelheim, Bayer, Eli Lilly, Novartis Pharmaceuticals, Regeneron, Pfizer, Roche Pharmaceuticals, Sanofi-aventis, Sanofi and Novo Nordisk outside the submitted work. NS reports grants and personal fees from Boehringer Ingelheim, Janssen, Novo Nordisk, Eli Lilly, Amgen, AstraZeneca and Sanofi outside the submitted work. No other potential conflicts of interest relevant to this article were reported.

## Abbreviations

AMI: Acute myocardial infarction
CVD: Cardiovascular disease
NHS: UK National Health Service
SCI-Diabetes: Scottish Care Information—Diabetes
SIMD: Scottish Index of Multiple Deprivation
SMR01: Scottish Morbidity Records
