# Supplementary material for: Trends in incidence and case fatality of acute myocardial infarction, angina and coronary revascularisation in people with and without type 2 diabetes in Scotland between 2006 and 2015
Source: Diabetologia. 2019 Jan 17;62(3):418–25. doi: 10.1007/s00125-018-4796-7 (PMC7019674; doi:10.1007/s00125-018-4796-7)
Supplement: Supplementary file 1 — (PDF 741 kb). [file 125_2018_4796_MOESM1_ESM.pdf]

Electronic Supplementary Material

TABLES:

ESM Table 1. Cameron and Trivedi test for over-dispersion

Coronary Heart Disease Type	Dispersion value	Z statistic	P-value
Acute myocardial infarction	1.34	25.08	<0.001
Angina	1.18	14.14	<0.001
Revascularisation	1.59	41.85	<0.001

ESM Table 2: Interaction coefficients for negative binomial models, adjusted for age, sex, diabetes status, deprivation and calendar year

**CHD Type**	**Interaction term**	**Relative risk [95% CI]**	**P-value**
**Myocardial Infarction**	Diabetes Type X Age	0.99 [0.98, 0.99]	<0.001
	Diabetes Type X Deprivation	1.00 [1.00, 1.01]	0.26
	Diabetes Type X Year	1.01 [1.00, 1.01]	<0.01
	Diabetes Type X Sex	1.29 [1.25, 1.33]	<0.001
	Age X Deprivation	1.00 [1.00, 1.00]	<0.001
	Age X Year	1.00 [1.00, 1.00]	<0.001
	Age X Sex	1.02 [1.02, 1.02]	<0.001
	Deprivation X Year	1.00 [1.00, 1.00]	0.99
	Deprivation X Sex	0.98 [0.97, 0.98]	<0.001
	Year X Sex	1.00 [1.00, 1.01]	0.39
**Angina**	Diabetes Type X Age	0.97 [0.97, 0.97]	<0.001
	Diabetes Type X Deprivation	1.00 [0.99, 1.01]	0.43
	Diabetes Type X Year	1.01 [1.00, 1.02]	<0.01
	Diabetes Type X Sex	1.15 [1.09, 1.22]	<0.001
	Age X Deprivation	1.00 [1.00, 1.00]	<0.001
	Age X Year	1.00 [1.00, 1.00]	0.45
	Age X Sex	1.01 [1.00, 1.01]	<0.001
	Deprivation X Year	1.00 [1.00, 1.00]	0.84
	Deprivation X Sex	0.97 [0.97, 0.98]	<0.001
	Year X Sex	1.00 [0.99, 1.01]	0.88
**Revascularisation**	Diabetes Type X Age	0.96 [0.96, 0.97]	<0.001
	Diabetes Type X Deprivation	1.01 [1.00, 1.02]	<0.01
	Diabetes Type X Year	1.00 [0.99, 1.01]	0.96
	Diabetes Type X Sex	1.41 [1.34, 1.49]	<0.001
	Age X Deprivation	1.00 [1.00, 1.00]	<0.001

Age X Year	1.00 [1.00, 1.00]	0.12
Age X Sex	1.01 [1.01, 1.01]	<0.001
Deprivation X Year	1.00 [1.00, 1.00]	0.14
Deprivation X Sex	0.95 [0.94, 0.96]	<0.001
Year X Sex	1.00 [0.99, 1.01]	0.67

ESM Table 3. Total number of coronary heart disease events and person-time by calendar year, sex and diabetes (events/person-time (1000s)).

CHD Type	Sex	Diabetes status	Overall	Total hosp person-time(1000s)	2006	2007	2008	2009	2010	2011	2012	2013	2014	2015
Myocardial infarction	M	No DM	65611	15579	6487/1507	6479/1520	6532/1531	6563/1541	6936/1552	6797/1567	6787/1575	6514/1584	6290/1595	6226/1604
	M	T2DM	15093	1146	1218/90	1317/96	1343/101	1488/107	1597/113	1545/117	1676/123	1614/128	1568/132	1727/136
	F	No DM	39925	17260	4398/1693	4092/1702	4126/1707	4123/1713	4184/1719	4030/1728	4101/1735	3774/1743	3616/1753	3481/1762
	F	T2DM	9297	929	875/76	778/80	847/84	924/88	948/92	999/95	955/98	1026/101	934/104	1011/106
Angina	M	No DM	18584	15603	2440/1509	2619/1522	2534/1533	2123/1543	1854/1555	1465/1570	1435/1578	1429/1587	1378/1597	1307/1606
	M	T2DM	4725	1152	531/90	572/96	560/101	468/107	505/113	384/118	378/123	445/129	426/134	456/137
	F	No DM	14809	17273	2002/1695	2064/1703	2081/1708	1664/1714	1444/1720	1153/1730	1158/1737	1157/1744	1099/1754	987/1763
	F	T2DM	3145	930	361/76	385/80	397/84	369/88	319/92	283/95	236/98	282/102	250/104	263/106
Revascularisation	M	No DM	42497	15591	4065/1508	4197/1521	4007/1532	4070/1542	4255/1553	4286/1569	4395/1576	4497/1585	4424/1595	4301/1605
	M	T2DM	8382	1138	685/90	763/96	723/101	783/106	818/112	828/117	903/121	912/126	969/131	998/134
	F	No DM	15770	17272	1572/1695	1635/1703	1544/1709	1479/1714	1546/1720	1574/1730	1617/1737	1628/1744	1628/1754	1547/1763
	F	T2DM	3226	930	295/76	268/80	258/84	337/88	316/92	338/95	287/98	361/101	376/104	390/106

M: Male, F: Females. No DM: No diabetes, T2DM: Type 2 diabetes

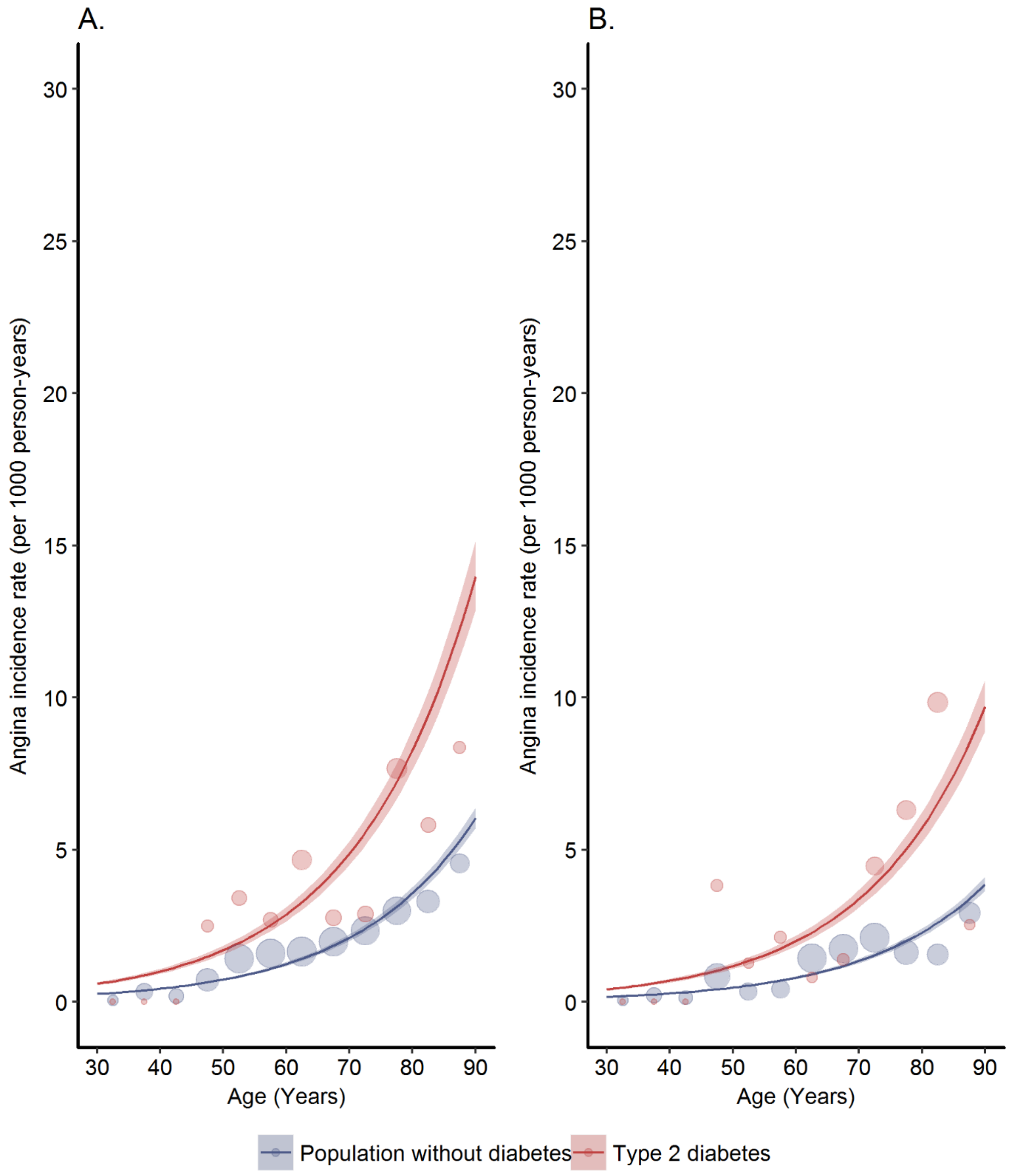

ESM Fig. 1. Incidence of angina by diabetes status and age for **A.** Men and **B.** Women. *Graph illustrates rates for people in deprivation decile 5 in the year 2010.* Model adjusted for age, sex, diabetes status, deprivation and calendar year. The shading shows 95% CIs.

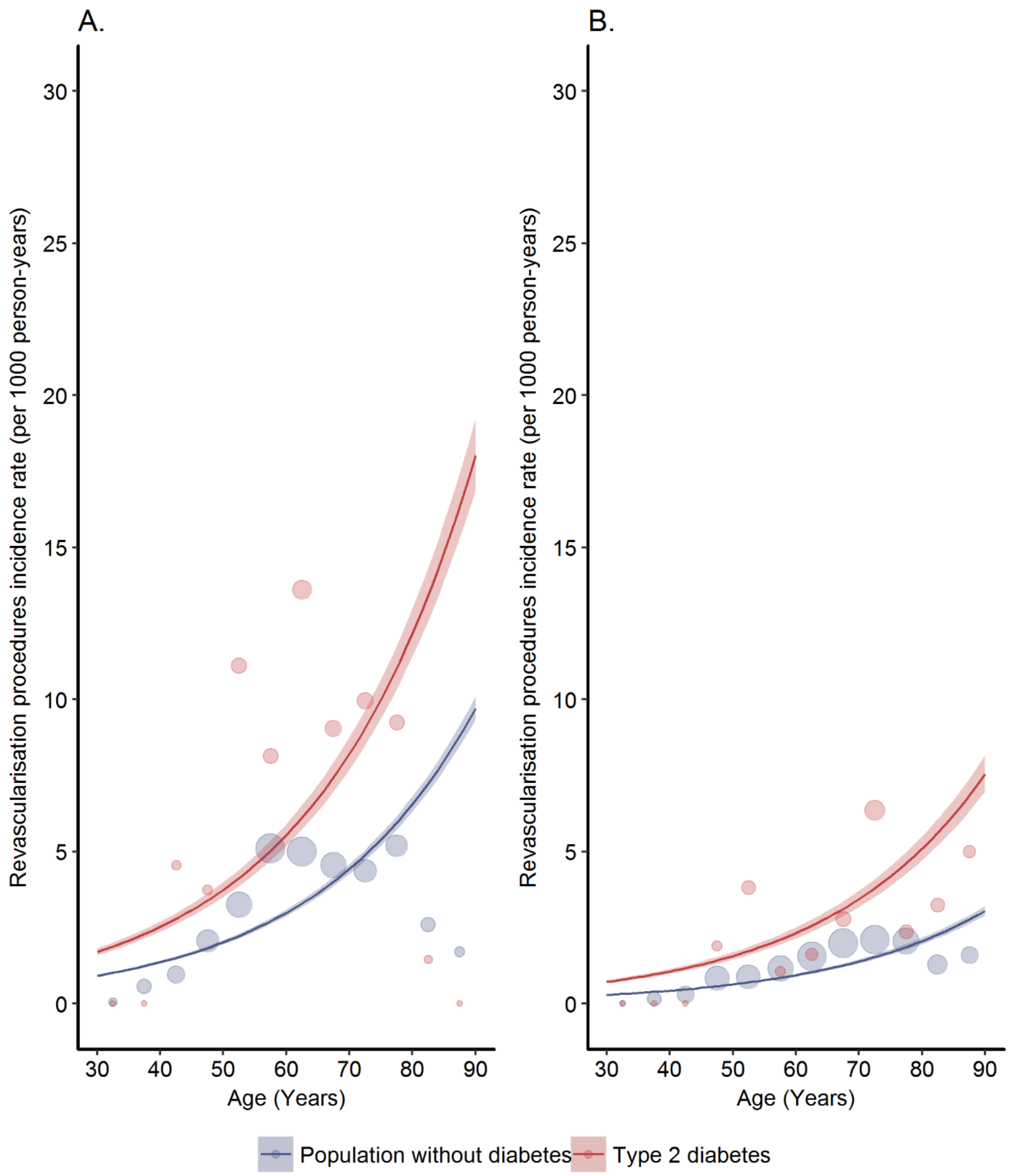

ESM Fig. 2. Incidence of revascularisation procedures by diabetes status and age for **A.** Men and **B.** Women. *Graph illustrates rates for people in deprivation decile 5 in the year 2010.* Model adjusted for age, sex, diabetes status, deprivation and calendar year. The shading shows 95% CIs.

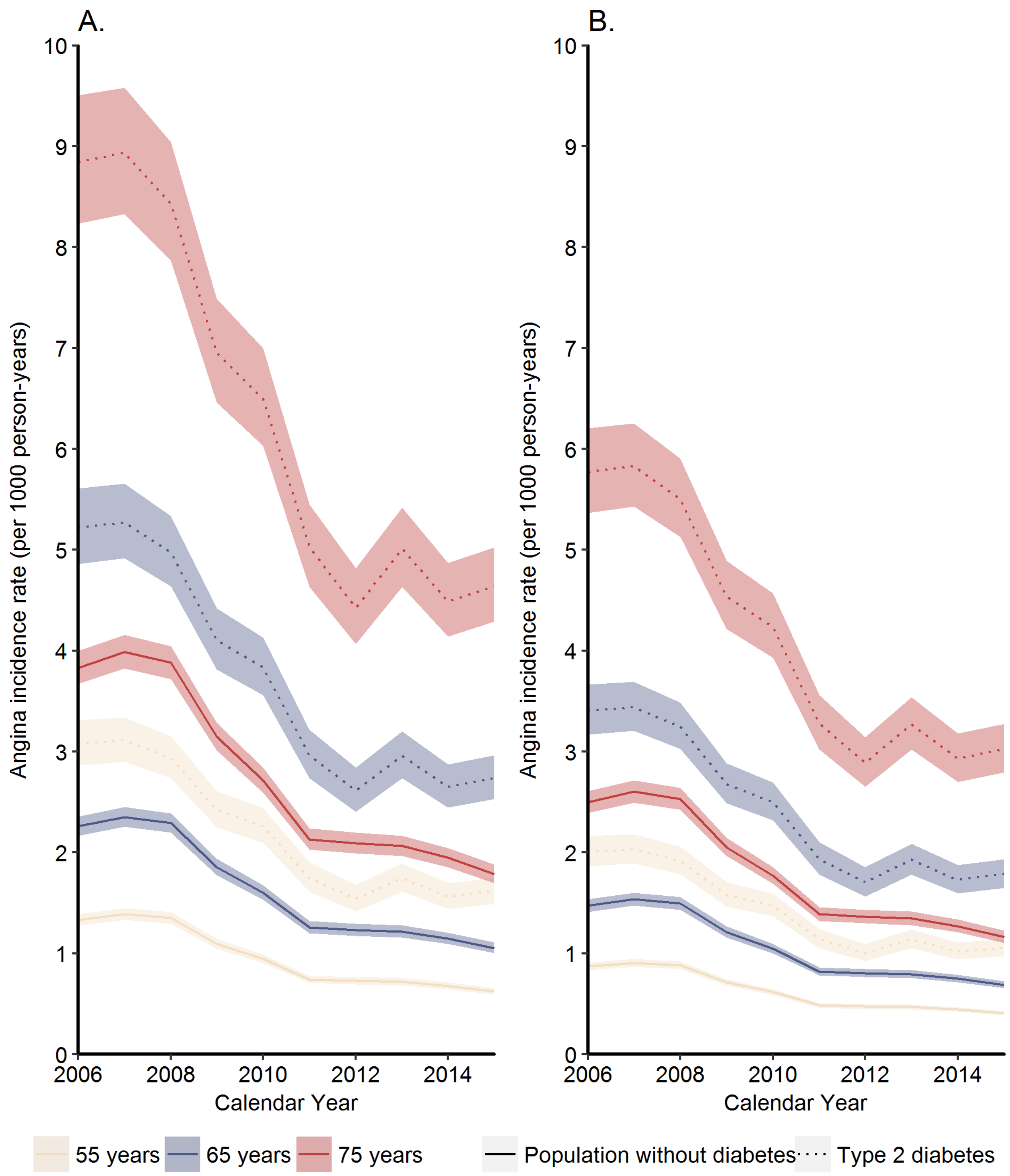

ESM Fig. 3. Incidence of angina by diabetes status and year for **A.** Men and **B.** Women. *Graph illustrates rates for people aged 55, 65 and 75 years and in deprivation decile 5.* Model adjusted for age, sex, diabetes status, deprivation, calendar year and interaction terms between sex and diabetes status, deprivation and diabetes status, diabetes status and year. The shading shows 95% CIs.

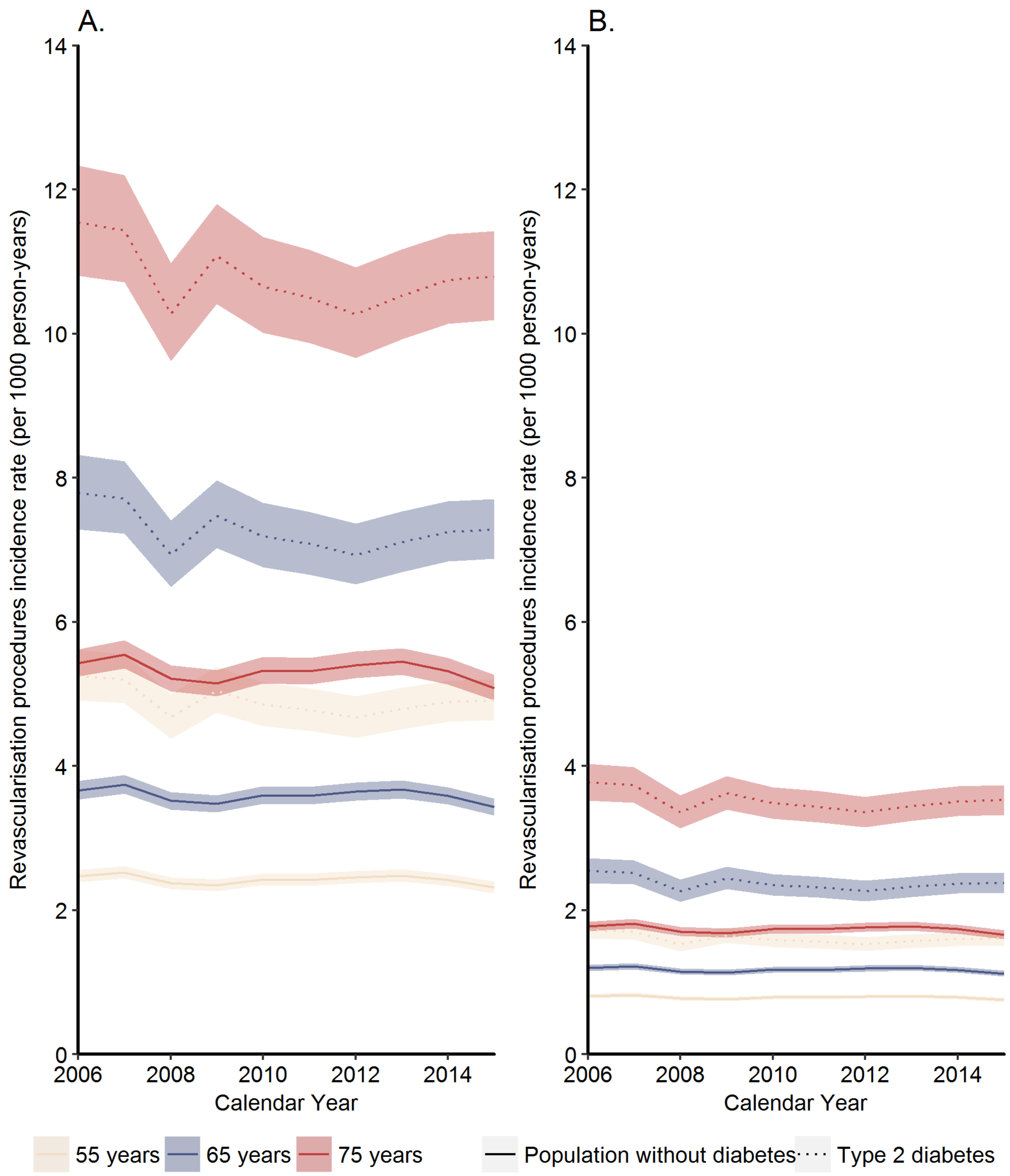

ESM Fig. 4. Incidence of revascularisation procedures by diabetes status, sex and year for **A.** Men and **B.** Women. *Graph illustrates rates for people aged 55, 65 and 75 years and in deprivation decile 5.* Model adjusted for age, sex, diabetes status, deprivation, calendar year and interaction terms between sex and diabetes status, deprivation and diabetes status, diabetes status and year. The shading shows 95% CIs.

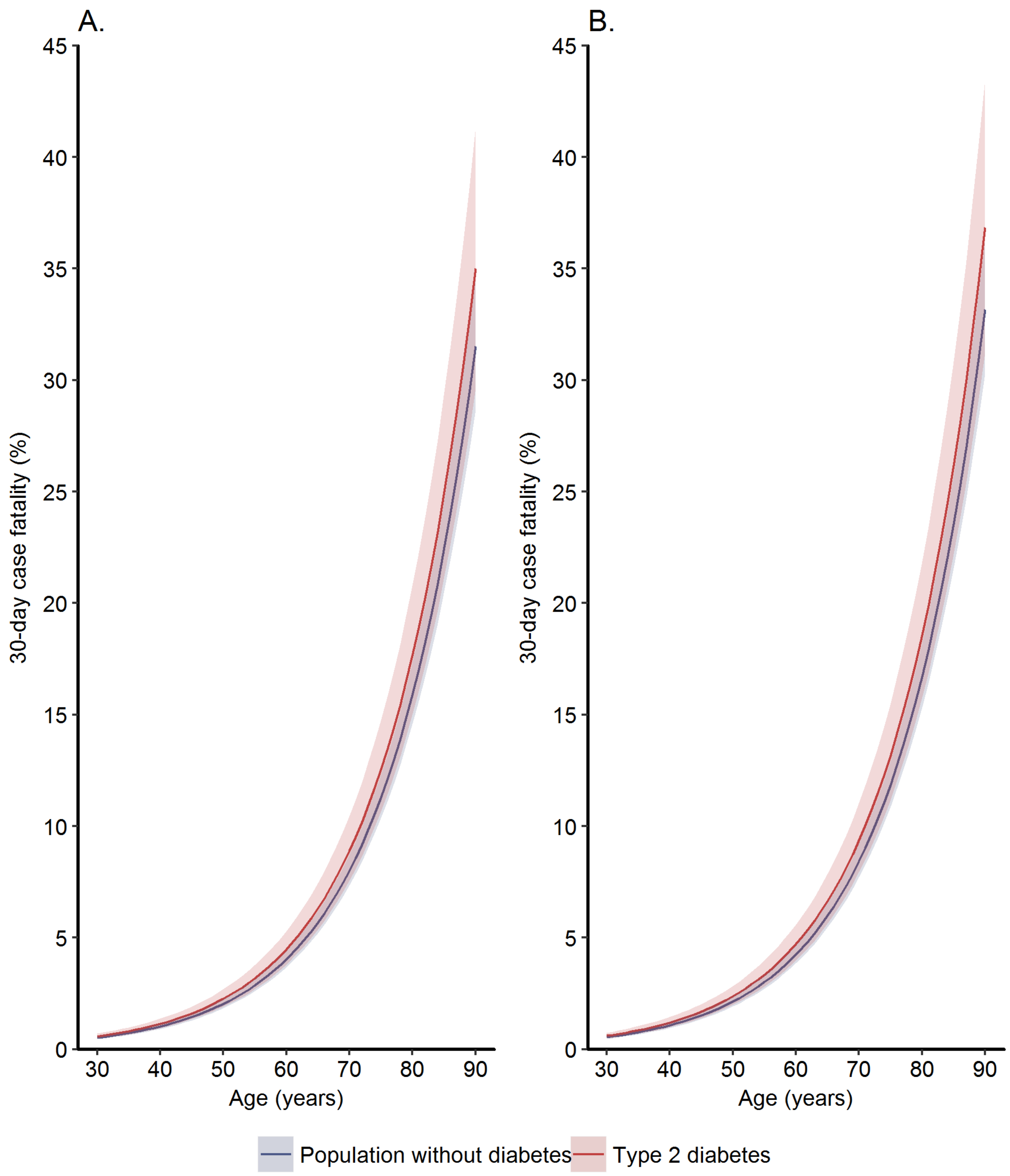

ESM Fig. 5. Case-fatality within 30 days of hospital admission following myocardial infarction by age, sex and diabetes status for **A.** Men and **B.** Women. Model adjusted for age, sex, diabetes status, deprivation, calendar year. The shading shows 95% CIs.
